# Laparoscopic Cholecystectomy Operative Time and Hospital Stay Differences Between Sicklers and Non-sicklers: A Five-Year Comparative Cross-Sectional Study at King Abdulaziz Medical City, Jeddah

**DOI:** 10.7759/cureus.30952

**Published:** 2022-10-31

**Authors:** Lamair A Albakri, Reem A Algarni, Rawan K Alrajhi, Yasmin A Yousef, Syed Faisal Zaidi

**Affiliations:** 1 Collage of Medicine, King Saud Bin Abdulaziz University for Health Sciences, Jeddah, SAU; 2 College of Medicine, King Abdullah International Medical Research Center, Jeddah, SAU; 3 Research and Development, King Abdullah International Medical Research Center, Jeddah, SAU; 4 College of Medicine, King Saud Bin Abdulaziz University for Health Sciences, Jeddah, SAU; 5 College of Medicine, King Saud Bin Abdulaziz University of Health Sciences, Jeddah, SAU; 6 Department of Surgery, Pediatric Surgery Section, King Abdulaziz Medical City, Jeddah, SAU; 7 Faculty of Eastern Medicine, Hamdard University, Islamabad Capital Territory, PAK

**Keywords:** cholelithiasis, cholecystitis, operative time, gallstones, laparoscopic cholecystectomy, sickle cell disease

## Abstract

Background

Sickle cell disease (SCD) is one of the most important hemoglobinopathies that result in the formation of pigment gallstones. Laparoscopic cholecystectomy (LC) is a safe surgical procedure for gallstones in SCD. Currently, there is no standard or guidelines for the preoperative preparation of these patients. This study aims to estimate the difference in pre-operative length of stay, operative time, postoperative length of stay, and total hospitalization length of stay among patients with and without SCD in a tertiary hospital in Jeddah, Saudi Arabia. Such knowledge would aid in establishing a standard for the preoperative preparation of SCD patients for LC.

Methods

Data from all patients undergoing laparoscopic cholecystectomy between January 2013 and December 2018 were collected retrospectively with a comparative cross-sectional study design. Data included age, sex, BMI, clinical presentation, mode of admission (elective or emergency), preoperative length of stay, operative time, postoperative length of stay, total hospitalization length of stay, and type of surgery (day or inpatient). JMP 15.2.1 was used for statistical analysis.

Results

From 2013 to 2018, 793 patients underwent LC, and of those, 16 (2.018%) were SCD patients. The results showed significant differences in preoperative (p<0.001), postoperative (p<0.001), and total hospitalization stay time (p<0.001) between the SCD patients and non-SCD patients. However, the data show no significant difference in the operative time of LC between the two studied groups.

Conclusion

SCD is the most common hemoglobinopathy-causing gallstone. Challenges in these patients are mainly in peri-operative management. Further prospective cohort studies are needed to create a standardized approach for peri-operative management of SCD patients to facilitate delivery of the same level of care and shorten total hospitalization time.

## Introduction

Sickle cell disease (SCD) is an autosomal recessive blood disorder associated with high morbidity and mortality. The prevalence of SCD varies among different parts of Saudi Arabia. The highest prevalence is in the eastern province, followed by the southwestern province. The reported prevalence of sickle cell trait in Saudi Arabia ranges from 2% to 27% depending on the region [1]. Patients with SCD have an abnormal form of protein (hemoglobin S). When hemoglobin-S gets deoxygenated, it forms rigid polymers that transform red blood cells (RBCs) into a sickle crescent shape. This leads to an increase in the viscosity and cell adhesion of RBCs, eventually resulting in vaso-occlusion [2]. Due to the hemolysis of sickled RBCs, an increased level of bilirubin excreted in the bile becomes a nidus for pigment stone formation. Thus, SCD patients experience a high incidence of gallstone formation [3].

Gallstones can be either symptomatic or asymptomatic. Symptomatic patients usually complain of pain in the right upper quadrant or epigastric region caused by biliary tree obstruction or cholecystitis, which can be acute or chronic [4]. Due to fewer postoperative complications, quicker recovery, better cosmetic postoperative outcomes, and less hospitalization time, laparoscopic cholecystectomy (LC) is considered the gold standard operative procedure for symptomatic cholelithiasis [5].

Based on the literature review, there was no established worldwide standard for the length of hospitalization and type of peri-operative management for SCD patients undergoing LC. Moreover, the rationale of the study is the lack of studies comparing SCD and non-SCD patients undergoing LC in Jeddah that might help in guiding the delivery of the same level of management in both groups. Therefore, this comparative cross-sectional study was designed to compare LC among patients with and without SCD in a tertiary care hospital in Jeddah, Saudi Arabia. The knowledge gained from this study estimates the difference in pre-operative length of stay, operative time, postoperative length of stay, and total hospitalization length of stay among patients with and without SCD in Jeddah. Such knowledge can be further directed to suggest a standard for preparing SCD patients for LC to ensure the safety of LC in such patients and to improve overall outcomes. This study provided a comparison of LC between SCD and non-SCD patients by estimating the difference in preoperative, operative, postoperative, and total hospitalization time.

## Materials and methods

A cross-sectional comparative study design was employed in this study. The data collection was done in the medical records accessed from paper files and an electronic health information system at a tertiary care facility in Jeddah, Saudi Arabia. The study included all patients that met the inclusion criteria from January 2013 to December 2018.

Patients aged 14 to 59 years old who had LC were included in the study. Pregnant women, patients who had other surgeries with LC, and patients with more than two comorbidities (hypertension, diabetes, hypothyroidism, etc.) were excluded. The total population of the study was 1409 patients. After applying the inclusion and exclusion criteria, only 793 patients were included. A consecutive sampling technique was used.

A data collection sheet was used to gather the information. Data were collected from the medical records by the researchers from the patients’ paper records and electronic health information systems. The variables collected in the data collection sheet were patient’s demographics (age, gender, height, weight, and clinical presentation), mode of admission (elective or emergency), preoperative length of stay, operative time, postoperative length of stay, total hospitalization length of stay, day or inpatient surgery, and co-morbidities.

Microsoft Excel program version 2019 (Microsoft® Corp., Redmond, WA) was used for data entry, and JMP 15.2.1 was used for statistical analysis. For descriptive statistics, the median was used to present quantitative variables such as age, while frequency and percentage were used to present qualitative variables. For inferential statistics, the Wilcoxon Rank Sums test was employed to compare quantitative variables, while the Chi-square and Fisher Exact tests were for comparing qualitative variables. A p-value <0.05 was taken as significant.

To maintain the confidentiality and anonymity of patient details, patient medical record numbers were collected and transferred to a separate coding sheet. Furthermore, information that may identify patients was not collected. Also, both hard and soft copies of the data were kept in a secure place that could be accessed by the research team only with the permission of the principal investigator. The data were stored in a password-protected file in a Microsoft Office Excel spreadsheet. The study was approved by the Institutional Review Board on January 6, 2020.

## Results

Table 1 shows the comparison characteristics of patients who had LC and met the inclusion criteria from 2013 to 2018. The total was 793 patients. Of these, 16 (2.018%) had SCD. Among 777 (97.982%) non-SCD patients, 190 (24.52%) were male and 585 (75.48%) were female, and their ages ranged from 15 to 59 years (median, 39 years). On the other hand, of the 16 SCD patients, eight (50%) were male and eight (50%) were female, and their ages ranged from 14 to 39 years (median, 21 years). Also, non-SCD patients had higher BMI (median, 30.092 kg/m²). The results show a significant age difference (p<0.001), gender (p<0.0355), and BMI (p<0.001) among the two studied groups.

**Table 1:** Comparison of characteristics between the studied groups

Items	Total LC Patients	Patients with no SCD*	Patient with SCD*	p-value
N (%)	793 (100%)	777 (97.982 %)	16 (2.018 %)	
Age				
Range	14–59	15–59	14–39	<0.001
Median	38	39	21	
Gender				
Male	198 (25.032%)	190 (24.52%)	8 (50%)	<0.0355
Female	593 (74.968%)	585 (75.48%)	8 (50%)	
BMI				
Range	14.992–53.277	15.823–53.277	14.992–29.474	<0.001
Median	29.903	30.091	22.36	
Presentation				
Acute cholecystitis	123 (15.51%)	118 (15.19%)	5 (31.25%)	
Chronic cholecystitis	484 (61.03%)	477 (61.39%)	7 (43.75%)	
Gallstone pancreatitis	18 (2.27%)	18 (2.32%)	0 (0%)	
Biliary colic	165 (20.8%)	161 (20.72%)	4 (25%)	
Obstructive jaundice	3 (0.39%)	3 (0.39%)	0 (0%)	

In comparing the modes of admission and presentation, 118 patients (15.19%) of non-SCD patients presented to the hospital with acute cholecystitis, 477 patients (61.39%) with chronic cholecystitis, and 45 patients (5.79%) with biliary colic. In contrast, five SCD patients (31.25%) presented to the hospital with acute cholecystitis, seven patients (43.75%) with chronic cholecystitis, and four patients (25%) with biliary colic.

In Table 2, 655 patients (84.30%) of non-SCD patients were admitted electively, and 122 patients (15.70%) were admitted as emergency cases. On the other hand, 13 patients (81.25%) with SCD were admitted electively, and three patients (18.75%) were admitted in an emergency. Of the non-SCD patients, 351 patients (45.17%) were operated on a day surgery basis, while all SCD patients had LC as inpatients (p<0.001).

**Table 2:** Comparison of mode of admission and type of surgery in the studied groups

Items	Total LC patients	Patients with No SCD*	Patient with SCD*	p-value
Mode of admission				
Elective	668 (84.24 %)	655 (84.30 %)	13 (81.25 %)	=0.7280
Emergency	125 (15.76 %)	122 (15.70 %)	3 (18.75 %)	
Type of surgery				
Day	351 (44.26 %)	351 (45.17 %)	0 (0%)	<0.001
Inpatient	442 (55.74 %)	426 (54.83 %)	16 (100%)	

Table 3 demonstrates a comparison of peri-operative time and total hospitalization time in the two studied groups. The results show that the pre-operative (p<0.001), post-operative time (p<0.001), and total hospitalization time (p<0.001) were longer in SCD patients. However, there was no significant difference in operative time (p=0.5175) between the two studied groups. As the data were collected retrospectively, a review of the operative notes was not always detailed enough to explain the intraoperative findings or difficulties.

**Table 3:** Comparison of peri-operative time and total hospitalization time in the studied groups

Items	Patients with no SCD*	Patients with SCD*	p-value
Preoperative time			
Range (hours)	0.266–266.33	40.733–200.5	<0.001
Median	10.55	82.258	
Operative time			
Range (min)	26–267	60–133	=0.5225
Median	91	102	
Postoperative time			
Range (hours)	0–144	15.466–127.416	<0.001
Median	14.85	31.7	
Total hospitalization time			
Range (hours)	2.73–348.883	72–329.333	<0.001
Median	34	120	

## Discussion

SCD is one of the more common hemoglobinopathies in Saudi Arabia. All hemoglobinopathies result in the formation of pigment gallstones due to increased heme component breakdown [1]. SCD is present in about 2% to 27% of the Saudi population depending on geographic distribution [1], with the highest prevalence in the Eastern region, followed by the Southwestern region. A review of the published literature showed that LC has proven to be a safe surgical procedure for the management of cholelithiasis in SCD patients and is not associated with an increase in major complications or mortality [6]. Al-Mulhim et al. conducted a retrospective study over five years from April 1994 until December 1998 in which they concluded that LC for acute cholecystitis in patients with SCD is safe and recommended because of the lack of significant complications. The same study further indicated that the complication rate was 17.5% and the mean hospital stay was 5.3 days [7]. Muroni et al. conducted a study from January 2000 to June 2014 to evaluate the effectiveness of prophylactic LC in SCD patients with asymptomatic gallbladder stones. The result of the study indicated that postoperative complications are less in asymptomatic patients who had a laparoscopic prophylactic cholecystectomy [8].

In this study, 2.018% of LC was performed in adults with SCD. This reported rate is considered low, especially in a country with a high prevalence of SCD [1]. This low number could be due to the high prevalence of cholelithiasis in children with SCD, which ranges from 30% to 70%. [3]. In addition, the calculated rate of SCD patients who had LC (2.018%) was found to be higher than the reported rate by Bonatsos et al. (0.31%) [9]. It must be emphasized that this study included only patients operated upon by general surgeons (>14 as per hospital policy). Pediatric patients (0-14 years) needing LC are operated upon by pediatric surgeons and have not been included in this study as they are a separate service.

Table 1 shows that the age range of non-SCD patients was 15-59 (median 39). However, SCD patients were younger, 14-39 (median 21), which is similar to what Meshikhes et al. and Dan et al. reported [10,11]. In this comparative cross-sectional study, among the non-SCD patients, the percentage of females was higher (75.48%). Similarly, a prospective study conducted by Meshikhes et al. reported a higher number of females in non-SCD patients (86%). Moreover, Meshikhes et al. reported that the percentage of females in SCD patients was higher (61%), while in this study the percentage of males and females was equal [10]. In addition, patients with non-SCD have a higher BMI (P<0.001). High BMI was classically labeled as a risk factor for gallstones [12]. On the other hand, SCD patients had gallstones due to their hemoglobin S and not due to high BMI, and this was confirmed as none of our patients had high BMI. Similar to what was reported by Al-Mulhim et al., chronic cholecystitis was found to be the most common presentation since 43.75% of SCD and 61.39 % of non-SCD patients presented with chronic cholecystitis. Acute cholecystitis (15.19%) was the second most common presentation in non-SCD, followed by biliary colic (14.93%), symptomatic cholelithiasis (5.79%), gallstone pancreatitis (2.32%), and obstructive jaundice (0.39%). In SCD patients, the percentage of acute cholecystitis was 31.25% and biliary colic was 25% [8].

As Table 2 shows, there is no association between the mode of admission and the diseased groups (p=0.7280). In this study, 81.25% (13 patients) of SCD patients had LC electively, while 18.75% (three patients) had emergency surgery. These results show similarities to the reported data of Plummer et al. in which 93% of the studied sample had elective LC and 6.25% had emergency surgery [13]. In addition, all SCD patients had LC as inpatients while 54.83% of non-SCD patients had inpatient surgery.

In Table 3, it was found that the median pre-operative time in SCD patients (82.258 hours) was longer than in non-SCD patients (10.55 hours). Also, the data indicate that there was no significant difference in the operative time between the two studied groups, and SCD patients showed surgery duration ranging from 60 to 133 minutes. In this regard, a study done by Plummer et al. showed a similar duration of surgery for SCD patients with a range of 70-150 minutes [13]. As the study was retrospective, limited details explaining the duration of the surgery were found in the operative reports. The results confirm that there is a significant difference in postoperative time and total hospitalization time between the two studied groups. The post-operative time of SCD patients ranged from 15.466 to 127.416 hours (0.64-5.31 days). Compared to what Plummer et al. reported, SCD patients required a one-to-thirteen-day postoperative hospitalization period [13]. Total hospitalization time was found to be longer in SCD patients, which ranged from 72 to 329.333 hours (3-13.72 days). This could be because they need more peri-operative management such as avoidance of dehydration, adequate oxygenation, peri-operative antibiotics, and blood transfusions to decrease their morbidity and mortality. A retrospective single-center study done by Al-Mulhim et al. at King Fahad Hospital, Hofuf, Saudi Arabia reported that LC in 427 adults with SCD had a short hospital stay with a mean hospital stay of 2.6 (range, 1-9) days [14].

This study helps in understanding the effect of SCD on management. The limitations of this study are the small number of SCD patients who had LC over 14 years and the limited number of studies comparing SCD and non-SCD patients. However, to the best of our knowledge, our study is the first study conducted to compare SCD patients and non-SCD patients in Jeddah and the first study to compare SCD and non-SCD patients in peri-operative time in Saudi Arabia. This study should be used as a basis for further prospective studies to try to establish a management algorithm or guidelines to optimize the outcome for SCD patients undergoing LC with the least necessary hospital stay and cost.

## Conclusions

In this study, the difference in peri-operative time of LC in SCD and non-SCD patients was calculated. Also, the preoperative, operative, postoperative, and total hospitalization times were compared between the two studied groups who underwent LC, and our statistical analysis concluded that patients with SCD have significantly longer preoperative, postoperative, and total hospitalization times compared with non-SCD patients. However, the data show no significance in the difference between the operative time of LC in the two studied groups. Further prospective studies are required to establish a standard for preparing SCD patients for LC. In addition, multicentric corroborative studies are needed in all regions of Saudi Arabia to study more SCD patients in the general population.
